# Changes in Biomass Carbon and Soil Organic Carbon Stocks following the Conversion from a Secondary Coniferous Forest to a Pine Plantation

**DOI:** 10.1371/journal.pone.0135946

**Published:** 2015-09-23

**Authors:** Shuaifeng Li, Jianrong Su, Wande Liu, Xuedong Lang, Xiaobo Huang, Chengxinzhuo Jia, Zhijun Zhang, Qing Tong

**Affiliations:** 1 Research Institute of Resource Insects, Chinese Academy of Forestry, Kunming, China; 2 The Pu`er Forest Eco-system Research Station, State Forestry Bureau, Kunming, China; 3 Forestry Research Institute of Pu’er Municipality, Pu’er, China; Tennessee State University, UNITED STATES

## Abstract

The objectives of this study were to estimate changes of tree carbon (C) and soil organic carbon (SOC) stock following a conversion in land use, an issue that has been only insufficiently addressed. For this study, we examined a chronosequence of 2 to 54-year-old *Pinus kesiya* var. *langbianensis* plantations that replaced the original secondary coniferous forest (SCF) in Southwest China due to clearing. C stocks considered here consisted of tree, understory, litter, and SOC (0–1 m). The results showed that tree C stocks ranged from 0.02±0.001 Mg C ha^-1^ to 141.43±5.29 Mg C ha^-1^, and increased gradually with the stand age. Accumulation of tree C stocks occurred in 20 years after reforestaion and C stock level recoverd to SCF. The maximum of understory C stock was found in a 5-year-old stand (6.74±0.7 Mg C ha^-1^) with 5.8 times that of SCF, thereafter, understory C stock decreased with the growth of plantation. Litter C stock had no difference excluding effects of prescribed burning. Tree C stock exhibited a significant decline in the 2, 5-year-old stand following the conversion to plantation, but later, increased until a steady state-level in the 20, 26-year-old stand. The SOC stocks ranged from 81.08±10.13 Mg C ha^-1^ to 160.38±17.96 Mg C ha^-1^. Reforestation significantly decreased SOC stocks of plantation in the 2-year-old stand which lost 42.29 Mg C ha^-1^ in the 1 m soil depth compared with SCF by reason of soil disturbance from sites preparation, but then subsequently recovered to SCF level. SOC stocks of SCF had no significant difference with other plantation. The surface profile (0–0.1 m) contained s higher SOC stocks than deeper soil depth. C stock associated with tree biomass represented a higher proportion than SOC stocks as stand development proceeded.

## Introduction

Forest ecosystem carbon (C) stock represents an important measure of the global C balance. C is predominantly stored in live biomass and soils, and to a smaller degree, in coarse woody debris [1]. Standing biomass C stock accounts for 82–86% of all aboveground C stock, while forest soils are estimated to contain about 73% of the global SOC stock [2–3]. Forests absorb atmospheric CO_2_ via photosynthesis, and subsequently, C contributions of soil C pool occur through decomposition of plant material (litter and root) [4]. Land-use changes have a significant impact on the global C balance by affecting the soil C accumulation rate and fine root turnover [5–6]. This in turn can alter vegetation biomass allocation and C stocks [7]. In most cases, a change of the land-use leads to increased CO_2_ emissions due to deforestation and decomposition of soil organic matter [8–9].

As one of the vital land-use types, reforestation may not only result in an increase of terrestrial C stock [10], but may also positively affect SOC sequestration due to the changing quality, quantity and temporal-spatial distribution of soil C inputs [11,12]. However, the impact of reforestation on soil C stock is not entirely clear and remains controversial. Previous studies have reached at least four different main viewpoints [3], stating that SOC stock is either a) increased [6,13,14], b) decreased [9,13–17], c) that changes in SOC stock are negligible [4,8], and d) that SOC stock level decreased initially to a significant degree, but then recovered to the original level [18]. Importantly, soil C is lost rapidly following deforestation during the process of conversion from primary forest to cropland, pasture and pine plantations [3,6,16], and may drop to 50% of the original level within the first 20 years following deforestation [19]. If anything, these different conclusions teach us that the factors affecting SOC sequestration are multivariate and include tree species, stand age, soil fertility, stand management measures, previous land use and climate [3,20,21,22].

Pine trees, regardless of exotic or native, are extensively planted on marginal or degraded lands that have inherently low soil fertility and SOC levels [17]. Most plantations are composed of pine trees in China, and forest biomass has sequestered 1.65±0.76 Pg C yr^-1^ since 1982 with a C accumulation rate of 57±26 g C m^-2^ yr^-1^ in standing tree biomass [10]. The increase of C accumulation has been primarily the result of reforestation [23]. The reforestation area accounts for 41% of the global reforestation in China and has increased by approximately 1.7 million ha yr^-1^ which directly translates into increased C stocks [22]. Nevertheless, soils represent the largest sink of terrestrial C, however estimates remain uncertain about the exact proportion [10]. In recent years, increasing attention has been paid to the changes in SOC following conversion from grassland or cropland, to shrub or forest [6,24,25], respectively. Despite these efforts, no studies have examined the changes in SOC stock levels associated with the conversion of secondary coniferous forests to *Pinus kesiya* var. *langbianensis* plantations, which is the focus of this study.

*P*. *kesiya* var. *langbianensis* is an important reforestation species that has been widely employed in the “Gain-for-green” program, which encompasses an area of 59.04×10^4^ ha and accounts for 3.71% of all woodlands in the Yunnan province [26,27]. For this reason, *P*. *kesiya* var. *langbianensis* has developed into an important source for resin and wood due to its rapid growth, excellent material quality and high resin production. The total area occupied by *P*. *kesiya* var. *langbianensis* plantation has steadily increased [27]. According to the National Forest Continuous Inventory, the proportion of plantation areas and standing stock increased from 2.86% to 10.57% and from 2.04% to 4.13% within the 20 years from 1987 to 2007 respectively [28]. As a main reforestation model in south Yunnan, Forest-to-pine plantation conversions can strongly impact C sequestration in tropical regions. Until recently, the lack of studies on the changes of C balance in the forest-to-pine plantations has made it difficult to provide precise estimates of how this affects vegetation biomass and soil C stocks. Our objectives were: (1) to quantify the C stock distribution of biomass and SOC following the conversion from secondary coniferous forests to *P*. *kesiya* var. *langbianensis* plantations based on a chronosequence (2, 5, 11, 13, 20, 26, 54-year-old plantation), and (2) to assess the changes in the size and contribution of these C stocks resulting from land-use changes that occurred during the conversion of a secondary coniferous forest to a *P*. *kesiya* var. *langbianensis* plantation.

## Materials and Methods

### Site description

The study was conducted in Pu’er city (23°3'-23°32' N, 100°28'-101°6' E) located south of Yunnan province, Southwest China, and the altitude ranged from 1500 m to 1800 m. This region’s climate is categorized as south subtropical mountain monsoon, which exhibits a distinct wet and dry season. The mean annual precipitation is 1490 mm, and more than 77% of rainfalls occur in the wet season (mostly from May to September), resulting in an average relative humidity ≥77%. The mean annual temperature is 17.6°C ranging from 11.4°C in January to 21.6°C in July. The soils are a tropical mountain red forest soil (China soil classification) that are highly fertile and retain above average levels of water.

### Current and past land use and stand management

*P*. *kesiya* var. *langbianensis* plantations, secondary coniferous forests and farmland were main land-use types in study region. According to the plantation manager, the dominant land-use conversion in Pu’er city were: (1) secondary coniferous forest to *P*. *kesiya* var. *langbianensis* plantation, (2) secondary coniferous forest to tea, coffee gardens or farmland. Together *P*. *kesiya* var. *langbianensis* plantation and secondary coniferous forest occupy more than 90% of woodlands, and trees of plantation display little difference in diameter at breast height (DBH) and height. Plantations were established in the remaining burned forest after clear-cutting secondary coniferous forest and extensive plantation began about 60 years ago. Selective cutting as housing and fuel in secondary coniferous forest was common style for local community the living for a long period in Pu’er city. In fact, reforestation in secondary coniferous forest was around near the town and contryside and stands had a certain extent disturbance. Stand management measures were similar to each other apart from the reforestation pattern in different stand ages. Seedling pots were the main afforestation method which occuried after 1998 with initial stand density of 2597 trees ha^-1^, seeding was predominantly used from 1980 to 1997, and aerial seeding started before 1980. Each plantation area is larger than 15 ha. In the first 7 years, weeding and fertilization helped establishing plantation, while 30% selective cutting intensity was common practice after that time as thinning interventions, in order to maintain the growth of *P*. *kesiya* var. *langbianensis* by increasing the radial growth of remaining trees and compensating for the reduced C sequestration of the tree layer [29]. Subsequently, selective cutting of some large trees (*DBH*≥24cm) took place in the mature forest including secondary coniferous forest. The herbaceous layer was dense in *P*. *kesiya* var. *langbianensis* plantation and dominant understory species commonly were *Breynia fruticosa*, *Melastoma polyanthum*, *Eupatorium adenphorum*, *Zingiber mioga* and *Selceria levis*.

### Fieldwork Permission

The project had been officially registered at the Research Institute of Resource Insects, Chinese Academy of Forestry and Yunnan Provincial Science and Technology Department. Pu’er forestry bureau issued the permission to conduct this study for all locations. The fieldwork did not involve endangered or protected species.

### Plot establishment

Based on the “National Forest Resource Continuous Investigation Technical Regulations”. *P*. *kesiya* var. *langbianensis* plantation chronosequences (2, 5, 11, 13, 20, 26 and 54-year-old) were selected in 2013 so that similar environmental conditions existed at all sites, which predominantly harbored low activity clays in soil depth of 0–0.3 m and medium activity clays below soil depth of 0.3 m. Secondary coniferous forests were located in the adjacent regions as a case control, for which stand age was about 60 years with trees now having reached *DBH* values sufficient for logging. Three replicates of different stand ages of *P*. *kesiya* var. *langbianensis* plantation and adjacent secondary coniferous forest were identified with the stand size exceeding 2 ha. Secondary coniferous forest can been divided into tree layers with *P*. *kesiya* var. *langbianensis* occupying in the upper of tree layer. Associated tree species of secondary coniferous forest were *Schima wallichii*, *Anneslea fragrans*, *Lithocarpus fenestratus*, *Castanopsis fleuryi*, *Castanopsis calathiformis*, *Machilus robusta*, *Eurya groffii*, *Wendlandia tinctoria* subsp. *intermedia*, *Helicia nilagirica*, *Myrica esculenta*, *Vaccinium exaristatum* and *Betula alnoides*. Most of these species occupied the sublayer of trees.

Three 20 m×20 m plots were randomly established within each stand age of chronosequence and secondary coniferous forest. Species names, *DBH* and height were documented for all trees (*DBH*≥1cm) within each plot. Stand characteristics are shown in Table 1.

**Table 1:** Stand characteristics of *Pinus kesiya* var. *langbianensis* plantations along stand age chronosequence and secondary coniferous forest.

Type	Age(yr)*	Location	DBH (cm)*	Height (m)*	Density (INS ha^-1^)*	Basal area of DBH (m^2^ ha^-1^)*
Plantation	2	23°28'N,100°29'E	1.09±0.12	1.33±0.01	1783±189	0.17±0.03
		23°29'N,100°28'E				
		23°28'N,100°30'E				
Plantation	5	23°29'N,100°29'E	6.46±0.36	4.63±0.37	1600±150	5.72±0.99
		23°30'N,100°29'E				
		23°30'N,100°28'E				
Plantation	11	23°29'N,100°32'E	13.81±0.53	10.9±0.28	900±50	14.91±1.48
		23°27'N,100°30'E				
		23°29'N,100°31'E				
Plantation	13	23°28'N,100°28'E	12.5±0.53	9.36±0.82	1200±156	15.3±1.24
		23°29'N,100°27'E				
		23°29'N,100°33'E				
Plantation	20	23°6'N,101°2'E	21.5±2.97	12.84±1.55	600±156	23.7±0.56
		23°8'N,101°9'E				
		23°10'N,101°16'E				
Plantation	26	23°3'N,101°6'E	19.84±1.51	16.77±0.21	816±227	26.21±3.42
		23°6'N,101°15'E				
		23°3'N,101°24'E				
Plantation	54	23°5′N, 101°2′E	30.41±2.42	17.93±1.89	508±104	37.39±2.5
		23°6′N, 101°18′E				
		23°9′N, 101°11′E				
Secondary coniferous forest	60	23°29′N, 100°32′E	7.97±0.37	7.96±0.28	2825±385	24.27±1.91
		23°28′N, 100°30′E				
		23°4′N, 100°5′E				

*:yr is year, stand mean ± whithin-stand standard errors(*SE*).

### Tree and understory C stock estimation

A total of 23 *P*. *kesiya* var. *langbianensis* trees were selected and harvested as sample trees. Biomass components were estimated individually by harvesting the trunk, branches, needles, cones and roots. Height was determined using a tape measure, and trunk was cut into 2 m sections and weighed for fresh mass, and a 5 cm thick disc was cut from the end of each stem section as a subsample. Roots were dug out manually and divided into stump, coarse roots (with a diameter ≥2 cm), large roots (with a diameter between 1 and 2 cm), medium roots (with a diameter between 0.5 and 1 cm), small roots (with a diameter between 0.2 and 0.5 cm) and fine roots (with a diameter ≤ 0.2 cm). subsamples of the trunk, branch, needle, cone and root from each sample tree were transported to the laboratory to determine the moisture content by oven-drying at 70°C to constant weight for dry biomass determination [30].

The dry biomass of each component was calculated from the dry/wet ratio of subsamples. Allometric equations were based on corresponding *DBH* and used to estimate biomass of pine components [21]. The models were based on the coefficient of determination (*R* ^2^) which explained more than 90% of the variability in trunk, branch, needle and root of *P*. *kesiya* var. *langbianensis* in our study and the standard error of estimation, the mean square residuals. Biomass allometric equations of broad leaf species in the secondary coniferous forest refered to Dang & Wu [31]. We used these equations estimate tree components biomass of the plantations and secondary coniferous forests (Table 2 and S1 Table).

**Table 2:** Allometric equations and carbon concentrations of each component of *Pinus kesiya* var. *langbianensis* and other broad leaf species.

Species types	Component	Allometric equation*	*R* ^2^	F-value	S.E.E^a^	MSR^b^	*P*	Carbon concentrations
*P*.*s kesiya* var. *langbianensis*	trunk	*Y* = 0.02*D* ^2.863^	0.984	409.136	0.205	0.042	<0.001	48.48
	branch	*y* = 0.007*D* ^2.757^	0.96	499.869	0.32	0.103	<0.001	48.13
	needle	*Y* = 0.151exp*D* ^0.175^	0.902	193.672	0.385	0.148	<0.001	47.27
	cone	*y* = -1.554+0.228*D*	0.577	28.678	1.309	1.712	<0.01	47.02
	root	*y* = 0.01*D* ^2.543^	0.973	758.294	0.24	0.058	<0.001	46.8
	total	*Y* = 0.043*D* ^2.755^	0.99	2174.815	0.154	0.024	<0.001	-
Evergreen oaks(Dang & Wu,1992)	trunk	*y* = 9.7566+1.4877E-02*D* ^3^	0.9899	-	–	–	–	0.5
	branch	*y* = 1.4497+6.9051E-03*D* ^3^	0.9764	–	–	–	–	0.5
	leaf	*y* = 3.01523E-02(-0.262+*D*)^2^	0.9627	–	–	–	–	0.5
	root	*y* = 6.5368E-05(8.2279+*D*)^4^	0.9931	–	–	–	–	0.5
Other evergreen species(Dang & Wu,1992)	trunk	*y* = 8.0443E-02*D* ^2.5142^	0.9693	–	–	–	–	0.5
	branch	*y* = 2.9416E-06(7.5074+*D*)^5^	0.9406	–	–	–	–	0.5
	leaf	*y* = 0.84424exp(0.1214*D*)-0.965	0.9630	–	–	–	–	0.5
	root	*y* = 7.1613E-05*(7.4892+*D*)^4^	0.9791	–	–	–	––	0.5
Deciduous species(Dang & Wu,1992)	trunk	*y* = 0.0237E-02(0.9067+*D*)^3^	0.9998	–	–	–	–	0.5
	branch	*y* = 1.9941+5.8425E-02*D* ^2^	0.9828	–	–	–	–	0.5
	leaf	*y* = -0.9807+0.4225*D*	0.9635	–	–	–	–	0.5
	root	*y* = 3.2221E-03(4.0749+*D*)^3^	0.9968	–	–	–	–	0.5

*:*y* is the biomass of the tree component (kg), *D* is the diameter at breast height (cm) where *a* and *b* are the equation parameters.

^a^S.E.E is the standard error of estimation

^b^MSR is the mean square residuals.

The understorey biomass including herbs, shrubs and tree seedling (*DBH*<1 cm) was measured in five 1m^2^ subplots that were located in the four corners and center of each plot (20 m×20 m). All above- and belowground live and dead plant biomass was harvested entirely, then transported to the laboratory and oven-dried at 70°C for biomass. C concentrations of trunk, branch, needle, cone and root of pine, as well as understory and litter were determined as described previously (Table 2), which were 48.48%, 48.13%, 47.27%,47.02%, 46.8%, 41.69% and 43.92% respectively [32,33] and conversion factor of broad leaf species was 0.5. Tree, understory and litter C stock (Mg C ha^-1^) were obtained by multiplying C concentrations by dry weight of each component.

### Soil sampling and laboratory analysis

Soil samples were randomly collected from the 24 plots of the eight stands in November 2013. Three soil profiles were dug for each plot with a soil depth of 1 m, following the removal of understory vegetation and litter. The soil profile was divided into five layers: 0–0.1, 0.1–0.2, 0.2–0.4, 0.4–0.6 and 0.6–1 m depth increments, and approximately 1 kg of soil were collected from every soil depth by compositing 5 subsamples in each plot. Soil bulk density for each soil layer was determined by volumetric rings (5 cm in diameter) obtained during the digging of soil subsamples.

The drying of volumetric rings subsamples occurred at 105°C to constant mass for determination of for bulk density and water moisture content.The other soil samples were air dried at room temperature (approximately 20°C), after removing plant roots, stones and debris which were sieved through a 2-mm mesh and stored in the laboratory at 4°C for detemination of soil charateristics. SOC concentrations were determined using the K_2_Cr_2_O_7_-H_2_SO_4_ titration method. Soil charateristics are summarized in Table 3 and S2 Table.

**Table 3:** SOC contents of *Pinus kesiya* var. *langbianensis* plantations along stand age chronosequence and secondary coniferous forest.

Stand types	Age (yr)	Soil depth (m)				
		0–0.1 (g kg^-1^)	0.1–0.2 (g kg^-1^)	0.2–0.4 (g kg^-1^)	0.4–0.6 (g kg^-1^)	0.6–1 (g kg^-1^)
plantation	2	20.26±5.62	8.58±1.7	5.86±0.7	3.43±0.42	2.67±0.41
plantation	5	32.47±8.33	15.22±0.91	10.77±2.41	5.94±1.69	3.82±0.78
plantation	11	38.96±2.09	22.39±2.96	15.64±0.77	11.34±1.54	6.45±0.67
plantation	13	42.1±10.16	24.58±6.02	14.98±2.84	9.13±1.03	6.35±1.34
plantation	20	19.68±1.6	13.73±0.32	9.9±0.11	5.64±0.42	3.96±0.21
plantation	26	22.5±1.34	16.04±2.07	12.55±1.95	8.09±1.32	5.46±1.07
plantation	54	36.82±2.08	27.86±2.85	20.13±2.04	11.95±2.09	11.53±2.35
SCF	60	27.54±4.1	17.59±3.47	11.25±2.3	5.94±0.32	4.58±0.81

SCF, secondary coniferous forest

Data are presented as the mean value±the standard errors

### Data calculation and analysis

The SOC stocks (Mg C ha^-1^) in each layer were calculated as follow:

$$SOC_{i}=0.1\times C_{i}\times B_{i}\times H_{i}\times (1-S_{i})$$

Where, *C*_i_ is the SOC concentration (g kg^-1^), *B*_i_ is the bulk density (g cm^-3^) and *H*_i_ is the thickness (cm), *S*_i_ represents the gravel content with a size >2mm of each soil layer. Total SOC stocks down to 1 m were calculated as the sum over all depth intervals.

The relative rate of change of SOC stock was used to evaluate the intensity of the SOC stock change. Each observed change in SOC stock (*R*_j_, Mg C ha^-1^ yr^-1^) after reforestation for the *j* interval was calculated as follows [3,24,25]:

$$R_{j}=\frac{C_{ej}-C_{cj}}{N}$$

where *C*_ej_ is the SOC stocks in the pine plantations (Mg C ha^-1^), *C*_cj_ is the SOC stocks of the *j* observation from the means of the secondary coniferous forest sites (Mg C ha^-1^), *N* is years after reforestation.

Normality of the data was determined using the Kolmogorov–Smirnov test. All data were log transformed when necessary to meet the assumption of normality. A one-way analysis of variance (ANOVA) was used to test the effect of stand age on the C stock in different tree components and soil depth. All assumptions of the ANOVA were fulfilled. Fisher’s least significant difference (LSD) test was performed for multiple post-hoc analyses to compare the tree C and SOC stock. All datas were statistically analyzed using SPSS17.0 (IBM, USA).

## Results

### Biomass C stocks

#### C stock allocation in pine plantation and secondary coniferous forest

Total tree C stock in all components of plantation increased with stand age up to 54 years (141.43±5.29 Mg C ha^-1^) when they were more than twice that of secondary coniferous forest (64.03±5.89 Mg C ha^-1^), 20-year-old (73.23±1.47 Mg C ha^-1^) and 26-year-old (70.48±2.23 Mg C ha^-1^) plantations. The tree C stock ranking of the plantation was identical between secondary coniferous forests and plantations: trunk > branch > root > needle > cone (Table 4). Cone C stock was negligible in 2-year-old stands. The understory C stock tend decrease with plantation agethrough age 13, and subsequently increased, but at a slower rate when compared to younger stands.

**Table 4:** Carbon stock allocation of all components of the trees, herbs and litter in the plantations and the secondary coniferous forest.

Stand age (yr)	Tree layer						Understory	Litter
	Trunk	Branch	Needle	Cone	Root	Total	(Mg C ha^-1^)	(Mg C ha^-1^)
	(Mg C ha^-1^)	(Mg C ha^-1^)	(Mg C ha^-1^)	(Mg C ha^-1^)	(Mg C ha^-1^)	(Mg C ha^-1^)		
2	0.15±0.01^f^	0.01±0.002^f^	0.02±0.01^e^	-	0.01±0.004^f^	0.20±0.001^e^	4.66±0.35^b^	-
5	3.98±0.41^e^	1.12±0.12^e^	0.37±0.03^d^	0.09±0.01^e^	1.01±0.18^e^	6.56±0.67^d^	6.71±0.70^a^	2.54±0.23^a^
11	20.48±1.31^d^	5.33±0.33^d^	0.91±0.06^d^	0.77±0.04^bc^	4.14±0.25^d^	31.64±1.99^c^	0.08±0.04^d^	1.71±0.11^b^
13	17.8±0.92^d^	4.69±0.24^d^	0.83±0.04^d^	0.72±0.02^c^	3.73±0.18^d^	27.78±1.40^c^	0.58±0.01^d^	1.99±0.24^b^
20	48.53±0.97^b^	11.91±0.34^b^	3.65±0.29^b^	0.91±0.03^b^	8.22±0.07^c^	73.23±1.47^b^	1.73±0.36^c^	2.15±0.34^ab^
26	46.65±1.45^b^	11.65±0.74^bc^	2.73±0.17^b^	1.12±0.08^a^	8.33±0.40^c^	70.48±2.23^b^	1.96±0.14^c^	2.67±0.32^a^
54	92.01±2.96^a^	22.11±1.2^a^	11.43±1.57^a^	1.27±0.08^a^	14.60±0.45^a^	141.43±5.29^a^	1.96±0.66^c^	2.38±0.19^ab^
SCF	39.74±3.13^c^	10.12 ±1.38^c^	3.39±0.52^b^	0.54±0.07^d^	10.24±0.91^b^	64.03±5.89^b^	1.15±0.18^cd^	2.78±0.31^a^

Data are presented as the mean value±the standard errors(SE) and in the table with the different letter in each row means difference significantly at *P*<0.05.

### C accumulation in standing biomass with stand development

A larger difference in accumulation rates of standing C stocks occurred in different growth stages (Table 5). With the growth of plantation, accumulation rates of all tree components rapidly increased up to 20 years (3.66 Mg C ha^-1^yr^-1^), after which slighty lower, but steady accumulation rates occurred. Conversely, accumulation rates of understory and litter C stock decreased with the increase of stand ages.

**Table 5:** Accumulation rates of standing biomass carbon stocks in different stand stages of *Pinus kesiya* var. *langbianensis* plantations.

	2 yr	5 yr	11yr	13 yr	20 yr	26 yr	54 yr
Type	Mg C ha^-1^yr^-1^	Mg C ha^-1^yr^-1^	Mg C ha^-1^yr^-1^	Mg C ha^-1^yr^-1^	Mg C ha^-1^yr^-1^	Mg C ha^-1^yr^-1^	Mg C ha^-1^yr^-1^
Trunk	0.01	0.8	1.86	1.37	2.43	1.79	1.7
Branch	0	0.22	0.48	0.36	0.6	0.45	0.41
Needle	0.08	0.07	0.08	0.06	0.18	0.1	0.21
Cone	0	0.02	0.07	0.06	0.05	0.04	0.02
Root	0.01	0.2	0.38	0.29	0.41	0.32	0.27
Sum tree	0.1	1.31	2.88	2.14	3.66	2.71	2.62
Understory	2.33	1.34	0.07	0.04	0.09	0.08	0.04
Litter	0	0.51	0.16	0.15	0.11	0.1	0.04

### SOC stock

#### Distribution of SOC stock

Profile distribution of the SOC stocks for the different stand ages are shown in Table 6. SOC stocks to 1 m increased up to age 11 and were variable thereafter, but remained above levels immediately after harvest. Secondary coniferous forest had a relatively constant SOC stock level of 123.37±14.59 Mg C ha^-1^ which was significantly higher than the 2-year-old stand. Reforestation resulted in losses of SOC stocks by an average of 42.29 Mg C ha^-1^ in the 2-year-old stand comparing secondary coniferous forest. Pine plantations at 54 years greatly exceeded this number by ~30%, since they reached up to 160.39±17.96 Mg C ha^-1^. However, the SOC stock level of younger plantations were either below (104.9±1.89 Mg C ha^-1^ in the 20-year-old stands) or above (150.76±2.34 Mg C ha^-1^ in the 13-year-old stands) the value we found in mature pine forest.

**Table 6:** SOC stocks contained in the 0–1 m soil layer measured at 0–0.1, 0.1–0.2, 0.2–0.4, 0.4–0.6, and 0.6–1 m soil depths under reforestation and secondary coniferous forest.

Depth (m)	Plantation (Mg C ha^-1^)							SCF
	2 yr	5 yr	11 yr	13 yr	20 yr	26 yr	54 yr	Mg C ha^-1^
0–0.1	26.12±5.12^ab^	32.37±6.57a^b^	33.97±2.35^ab^	37.14±5.93^a^	22.51±1.51^b^	27.04±1.02a^b^	31.16±1.38^ab^	30.6±3.14^ab^
0.1–0.2	11.96±2.22^c^	17.14±1.22b^c^	22.25±2.77^ab^	24.44±4.37^ab^	18.47±1.08^abc^	19.64±2.85a^bc^	26.04±3.28^a^	20±3.12^abc^
0.2–0.4	16.3±1.72^c^	26.62±6.01a^bc^	33.34±2.61^ab^	34.05±5.09^ab^	26.59±1.47^abc^	30.15±5.27^ab^	37.42±3.31^a^	28.32±4.4^abc^
0.4–0.6	10.46±1.23^c^	15.53±4.44^bc^	26.91±4.5^a^	22.81±3.17^ab^	15.32±0.54^bc^	18.3±2.76^abc^	22.42±3.44^ab^	17.29±0.72^bc^
0.6–1	16.23±2.52^c^	21.25±4.87bc	30.79±3.94^abc^	32.34±8.16^ab^	21.9±0.47^bc^	25.53±4.2^bc^	46.35±6.86^a^	27.17±4.75^bc^
0–1	81.08±10.13^d^	112.91±22.11^bcd^	147.26±8.69^ab^	150.76±2.34^ab^	104.9±1.89^cd^	120.66±15.5^abcd^	160.39±17.96^a^	123.37±14.59^abc^

Data are presented as the mean value±the standard errors(SE) and in the table with the different letter in each row means difference significantly at *P*<0.05.

SOC stocks in the 0–0.1 m soil depth of 5-year-old, 11-year-old and 13-year-old were higher than those of middle-aged and older stands as well as secondary coniferous forest. SOC stocks contained in the 0.2–0.4 m soil depth were largest in 11-year-old, 13-year-old and 54-year-old, and the highest SOC stocks were found in the 0.6–1 m soil depth, which were measured in the 54-year-old. In 2, 5 and 20-year old plantations, there was lower SOC stock contained in each soil depth compared with the secondary coniferous forest, whereas there were greater SOC stocks in the 11, 13 and 54-year-old, and similar amounts in the 26-year-old compared to secondary coniferous forest.

#### Changes in SOC stock after reforestation

**Fig 1:**
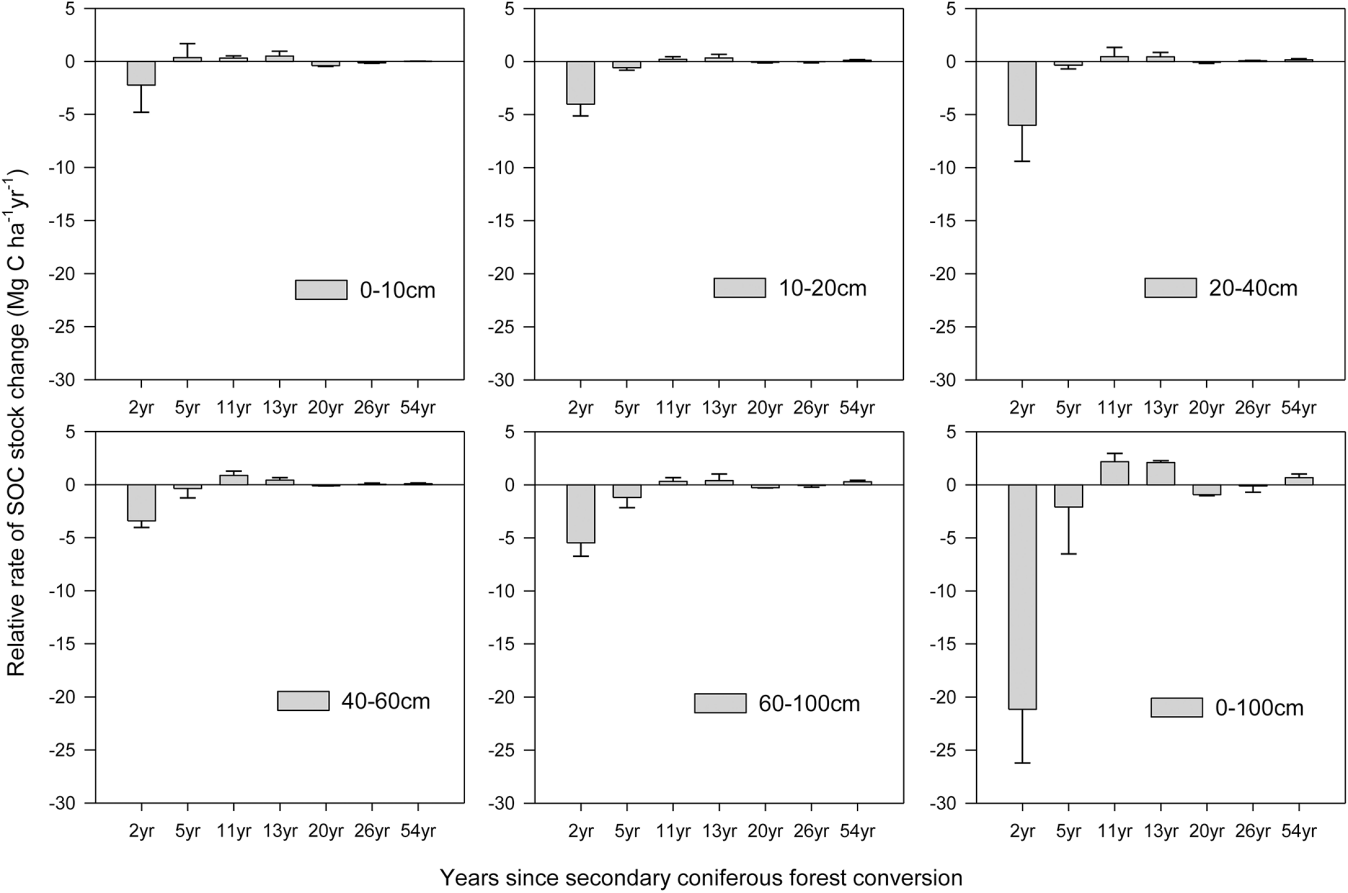
Changes in the SOC stocks contained in different soil layers in response to the length of time after converting secondary coniferous forest to pine plantation.

The accumulation of SOC stock over a soil depth of 0–1 m changed following the conversion of the secondary coniferous forest to the pine plantation (Fig 1). The SOC stocks were significantly reduced by 21.14±5.06 Mg C ha^-1^ yr^-1^ during the two years after reforestation, and the rates of SOC change were greater in the surface soil (especially at 0–0.1 m soil depth, -0.24±2.56 Mg C ha^-1^ yr^-1^) than in deeper soil depth (0.1–1 m). After reforestation, the rates of change in the SOC stocks contained in the surface soil (0–0.1 m) differed from deeper soil (0.1–0.2 m, 0.2–0.4 m, 0.4–0.6 cm and 0.6–1 m), and the ability of deeper soil layer for SOC sequestration lagged behind that of the surface soil. However, at depths of 0.1–0.2, 0.2–0.4, 0.4–0.6 and 0.6–1 m, SOC stocks showed the same change. After the secondary coniferous forest had been converted to the pine plantation, the SOC stock of 11 to 20-year-old stands contained in the 0–0.1 m and 0–1 m had little diference.

### Proportion of tree C and SOC stocks in plantation and secondary coniferous forest

**Fig 2:**
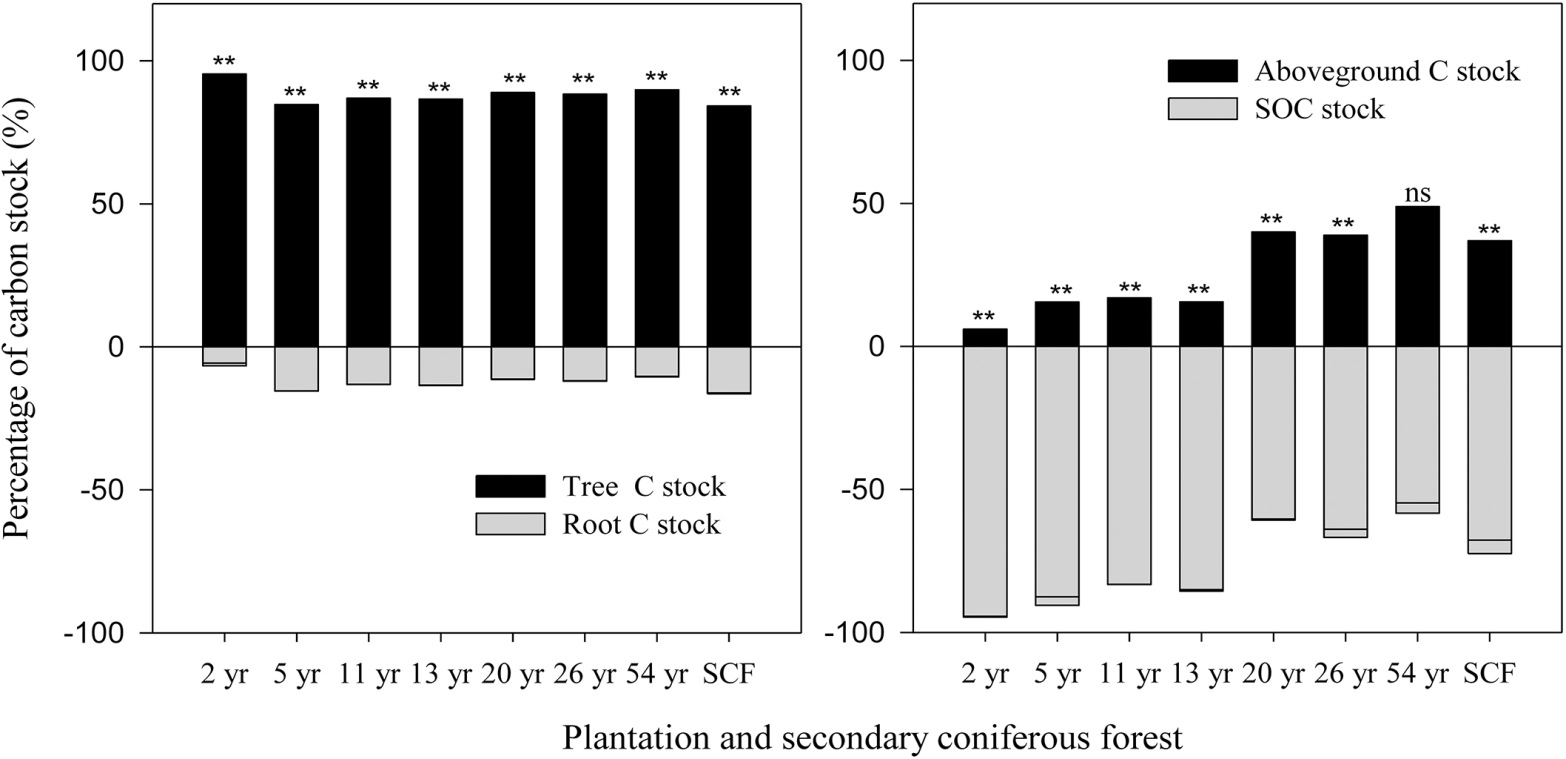
The proportion of aboveground and root carbon stock, tree carbon and SOC stock along stand age chronosequence and secondary coniferous forest. ns, no significance;*,** significant difference at the 0.05 and 0.01 level respectively.

As shown in Fig 2, the proportion of aboveground C stock was larger than root C stock, with corresponding values of 94.38% and 5.62%, respectively in 2-year-old stands. The proportion of tree C stock held aboveground and in roots ranged from 84.66±0.05% to 94.38±0.98% and from 5.64±0.98% to 15.34±0.05%, dependent on the age of the stands. SOC stock was the main C source in the early stand stages, but slowly decreased over time in importance relative to tree C stock, in particular SOC stock contained 52.83±3.52% of the total C stock in 54-year-old stands, after which tree C stock gradually became important component of C sequestration.

The ratio of aboveground to root C stock was not significantly correlated with stand age (*r* = -0.19, *P* = 0.41, n = 21), whereas the ratio of tree C to SOC stocks was positively correlated with stand age (*r* = 0.872, *P*<0.001, n = 21).

## Discussion

### Biomass C and SOC stock

Reforestation is one of the main processes responsible for regional C uptake in the southern regions of China [10]. We showed here that reforestation after clearing of secondary coniferous forest results in an increase of standing biomass C and SOC stock. The tree C and SOC stocks following the conversion from a secondary coniferous forest to a pine plantation gradually accumulated as stands grew and matured. Our study results were consistent with previous studies on the dynamics of tree C stock in young and middle-aged pine plantations [33,34]. Tree C and SOC stocks ranged from 0.02±0.001 Mg C ha^-1^ to 141.43±5.29 Mg C ha^-1^ and 81.08±10.13 Mg C ha^-1^ to 160.39±17.96 Mg C ha^-1^, respectively. Nearly mature- and mature plantations of *P*. *kesiya* var. *langbianensis* had significantly higher tree C and SOC stock than warm temperate coniferous forests and tropical forests in China [35]. However, biomass C of pine plantation was less than that of tropical forests (157 Mg C ha^-1^) found outside China [19]. At the same time, however, SOC stocks of 13 and 54-year-old *P*. *kesiya* var. *langbianensis* plantations were higher than those found in 45-year-old *P*. *yunnanensis* plantations [14].

*P*. *kesiya* var. *langbianensis* is a fast-growing tree species that can produce high yields in a short period [27], and which has a minimum rotation time of 51 years [26]. Our results showed that C accumualtion in the tree C and SOC stocks of pine plantations were able recover to the level of adjacent secondary coniferous forests after 20 and 11 years, respectively. Reforestation age was an important factor that impacted tree C accumulation after conversion, and the ratio of tree C to SOC stocks was positively correlated with stand age. The finding was in accordance with other previous studies on pine forests [21]. The contribution of tree C stock to the total C stock increased almost 200 fold from 0.20±0.001 Mg C ha^-1^ in the 2-year-old stand to 141.43±5.29 Mg C ha^-1^ in the 54-year-old stand. Our data therefore suggests that young pine plantations, which are predominantly found in the south and southwest of Yunnan, exhibit a high degree of C sequestration potential.

However, a constant increase in the production of forest biomass may not necessarily result in an increase of SOC stock [19]. Generally, SOC stock initially declines following the conversion of a secondary coniferous forest to a pine plantation. Previous studies have shown that whole-tree harvesting causes a decrease in soil C stocks by approximately 6% [20], but these losses are regained afterwards as stands mature [12,17]. These results are consistent with our observations from the first 2 years following pine reforestation which SOC stocks decreased by 42.29% compared to secondary coniferous forest, and thereafter SOC stocks did not represent a net gain but rather a recovery of C loss as a result of reforestation [11]. SOC stocks accumulated significantly in the 11, 13 and 54-year-old stands, a finding that was consistent with mature stand of *P*. *yunnanensis* plantations [14].

### Change in SOC stock

Studies examining the relative rate of C stock change usually included a comprehensive study of SOC stock following land use [3,24,25], which is considered a better way to describe the effects on land use change on C stocks in terrestrial ecosystems. SOC accumulation was significantly and positively correlated with stand age in the early stages of the forest-to-pine plantation conversion. This demonstrated that above- and belowground C stocks decreased markedly after reforestation, but slowly increased afterwards. A previous study showed that the net productivity of *P*. *kesiya* reached a peak in 5 to 7-year-old stands in India, which was followed by a distinct decline in older pine plantations [34]. Several studies have reported a decrease in SOC following the replacement of native vegetation by *Pinus* in Brazil [36]. The net effects of changes in total tree C and SOC stock were not only dependent on new C input, but also on C lost from the previous management regime [37]. SOC loss was highest in the earlier stages of the plantations in relation to soil disturbance resulting from clear-cutting harvesting and site preparation in the process of reforestation [19]. Subsequent the demand on soil nutrients by aboveground vegetation growth after reforestation results in C accumulation mainly in the aboveground vegetation.

SOC accumulation in the early stages of stand development depends on the stand and reforestation management operations. Quantity of C inputs accompanied by new increased organic matter promotes SOC accumulation [24]. SOC accumulation in both topsoil and subsoil varies with the time following reforestation. Stand management could strongly influence surface vegetation and soil nutrients by limiting the amount of litter returned to the soil thus facilitating the loss of top-soil [6]. After clear-cutting of secondary coniferous forest, soil moisture generally decreases at the surface but increases in lower soil depths (due to less plant water use), leading to greater decomposition in deeper soil depths (and overall, due to the combined effects of overall higher soil moisture and warmer temperatures) [38]. SOC accumulation in deep soil depth should be taken into account when assessing the total SOC change of the soil profile in long-term study [25]. Another reason that may account for SOC accumulation of deeper soil layers is that vegetation cover was sparse in young plantations, which reduced the soil C input and may have altered the microclimatic conditions, thus resulting in increased soil organic matter decomposition rates.

Positive accumulation of SOC stocks in middle-aged and over-mature pine plantations may be explained by the interactions between factors such as stand density, canopy, understory vegetation and soil properties [14] and thinning interventions [29]. The near-mature stands (including in 20, 26-year-old) were often not managed appropriately (such as stopping designed seletive cutting and understory tending), which impacts the net increase in SOC. *P*. *kesiya* var. *langbianensis* grew well in middle-aged plantations (including in 11, 13-year-old) with higer litter input and corresponding SOC stocks due to thinning interventions residues into the soil compensated for C losses. Self-thinning and the quantity of dead branches and fallen leaves increased as stand age increased. The mature pine plantations displayed a healthy range of understory species, high nitrogen stock, water moisture, tree and litter biomass. Increasing forest productivity can enhance the C storage capacity of the stable pool and thereby increasing the C input to the soil [29], so older forests (54-year-old plantation) may eventually store more SOC stocks.

## Supporting Information

S1 Table DBH, height and biomass of each component of 23 *Pinus kesiya* var. *langbianensis* sample tree. (DOCX)

S2 Table Site and characteristics of the plantation and secondary coniferous foeret plots. (DOCX)
