# Supplementary material for: Changes in Biomass Carbon and Soil Organic Carbon Stocks following the Conversion from a Secondary Coniferous Forest to a Pine Plantation
Source: PLoS One. 2015 Sep 23;10(9):e0135946. doi: 10.1371/journal.pone.0135946 (PMC4580575; doi:10.1371/journal.pone.0135946)
Supplement: S1 Table — (DOCX) [file pone.0135946.s001.docx]

**Tabel S1 DBH, height and biomass of each component of 23 *Pinus kesiya* var. *langbianensis* sample tree**

| Tree number | DBH (cm) | Height (m) | Biomass (kg) | | | | | |
| --- | --- | --- | --- | --- | --- | --- | --- | --- |
|  |  |  | trunk | branch | needle | root | cone | total |
| 1 | 4.1 | 2.2 | 0.93 | 0.48 | 0.32 | 0.53 | 0.00 | 2.27 |
| 2 | 4.9 | 3.86 | 2.10 | 0.50 | 0.38 | 0.52 | 0.00 | 3.50 |
| 3 | 5.2 | 3.42 | 2.26 | 0.76 | 0.37 | 0.71 | 0.00 | 4.11 |
| 4 | 5.3 | 3.9 | 2.90 | 0.89 | 0.37 | 0.94 | 0.00 | 5.10 |
| 5 | 6.4 | 3.55 | 3.85 | 1.83 | 0.89 | 1.53 | 0.00 | 8.11 |
| 6 | 7.9 | 9.6 | 9.08 | 1.18 | 0.39 | 1.30 | 0.00 | 11.95 |
| 7 | 8.3 | 9.5 | 9.51 | 1.72 | 0.53 | 1.50 | 0.00 | 13.26 |
| 8 | 9.3 | 9 | 12.29 | 2.66 | 0.53 | 2.70 | 0.29 | 18.47 |
| 9 | 9.7 | 10.8 | 15.94 | 2.33 | 0.70 | 2.74 | 0.00 | 21.70 |
| 10 | 10.8 | 10.95 | 16.87 | 3.23 | 0.55 | 3.11 | 0.00 | 23.76 |
| 11 | 13.4 | 12.37 | 26.55 | 6.07 | 1.85 | 7.14 | 0.47 | 42.08 |
| 12 | 14.4 | 11.7 | 37.13 | 12.36 | 1.52 | 6.75 | 1.72 | 59.48 |
| 13 | 14.5 | 12.5 | 41.85 | 14.17 | 1.76 | 10.97 | 1.67 | 70.41 |
| 14 | 14.5 | 10.9 | 38.10 | 16.48 | 3.16 | 10.02 | 0.80 | 68.55 |
| 15 | 15.1 | 12.35 | 49.25 | 16.48 | 2.63 | 12.99 | 1.09 | 82.44 |
| 16 | 17.4 | 13.33 | 55.34 | 19.18 | 2.75 | 15.51 | 4.26 | 97.05 |
| 17 | 18 | 12.9 | 62.13 | 30.74 | 9.23 | 16.55 | 5.62 | 124.27 |
| 18 | 18.3 | 13.9 | 81.56 | 19.94 | 5.08 | 14.71 | 0.00 | 121.28 |
| 19 | 18.4 | 12.8 | 57.19 | 22.47 | 2.69 | 13.62 | 2.50 | 98.48 |
| 20 | 19.3 | 12.7 | 78.58 | 33.98 | 6.28 | 20.43 | 4.73 | 144.00 |
| 21 | 23.5 | 19.8 | 231.26 | 35.42 | 7.40 | 40.00 | 5.88 | 319.96 |
| 22 | 24.8 | 19.3 | 290.04 | 58.84 | 7.23 | 49.35 | 2.59 | 408.05 |
| 23 | 25.6 | 19.3 | 278.63 | 41.24 | 12.04 | 36.06 | 3.20 | 371.18 |
