# Supplementary material for: Changes in Biomass Carbon and Soil Organic Carbon Stocks following the Conversion from a Secondary Coniferous Forest to a Pine Plantation
Source: PLoS One. 2015 Sep 23;10(9):e0135946. doi: 10.1371/journal.pone.0135946 (PMC4580575; doi:10.1371/journal.pone.0135946)
Supplement: S2 Table — (DOCX) [file pone.0135946.s002.docx]

**Table S2 Site and characteristics of the plantation and secondary coniferous foeret plots.**

| Stand types | CL. | Age (yr) | alt.(m) | Depth(m) | Soil moisture (%) | Bulk density (g/cm^-3^) | pH | SOC (g/kg) | Total nitrogen (g/kg) | Available P（mg/kg) | C:N |
| --- | --- | --- | --- | --- | --- | --- | --- | --- | --- | --- | --- |
| plantation | 7 | 2 | 1650 | 0-0.1 | 20.94 | 1.15 | 5.22 | 31.38 | 1.09 | 2.44 | 28.84 |
| plantation | 7 | 2 | 1650 | 0.1-0.2 | 19.74 | 1.39 | 5.69 | 8.98 | 0.60 | 1.02 | 14.85 |
| plantation | 7 | 2 | 1650 | 0.2-0.4 | 20.82 | 1.32 | 5.25 | 6.17 | 0.47 | 1.28 | 13.16 |
| plantation | 7 | 2 | 1650 | 0.4-0.6 | 17.08 | 1.50 | 5.87 | 3.10 | 0.34 | 1.33 | 9.01 |
| plantation | 7 | 2 | 1650 | 0.6-1 | 20.74 | 1.52 | 5.17 | 2.81 | 0.30 | 0.92 | 9.38 |
| plantation | 8 | 2 | 1660 | 0-0.1 | 14.01 | 1.42 | 5.38 | 13.34 | 0.64 | 1.83 | 20.71 |
| plantation | 8 | 2 | 1660 | 0.1-0.2 | 17.61 | 1.44 | 5.32 | 5.46 | 0.34 | 0.91 | 16.04 |
| plantation | 8 | 2 | 1660 | 0.2-0.4 | 18.87 | 1.47 | 5.33 | 4.52 | 0.34 | 0.73 | 13.48 |
| plantation | 8 | 2 | 1660 | 0.4-0.6 | 21.61 | 1.56 | 5.44 | 2.93 | 0.31 | 0.60 | 9.45 |
| plantation | 8 | 2 | 1660 | 0.6-1 | 19.88 | 1.52 | 5.41 | 1.89 | 0.23 | 0.57 | 8.09 |
| plantation | 9 | 2 | 1680 | 0-0.1 | 13.88 | 1.45 | 5.44 | 16.05 | 0.73 | 2.20 | 21.85 |
| plantation | 9 | 2 | 1680 | 0.1-0.2 | 15.54 | 1.37 | 5.85 | 11.31 | 0.61 | 1.57 | 18.49 |
| plantation | 9 | 2 | 1680 | 0.2-0.4 | 19.42 | 1.40 | 5.65 | 6.89 | 0.52 | 1.02 | 13.30 |
| plantation | 9 | 2 | 1680 | 0.4-0.6 | 20.75 | 1.51 | 5.68 | 4.27 | 0.33 | 0.79 | 12.84 |
| plantation | 9 | 2 | 1680 | 0.6-1 | 21.18 | 1.53 | 5.68 | 3.30 | 0.35 | 0.66 | 9.50 |
| plantation | 1 | 5 | 1700 | 0-0.1 | 19.55 | 1.13 | 5.29 | 24.74 | 0.78 | 4.23 | 31.52 |
| plantation | 1 | 5 | 1700 | 0.1-0.2 | 21.51 | 1.21 | 5.34 | 14.62 | 0.54 | 5.84 | 26.89 |
| plantation | 1 | 5 | 1700 | 0.2-0.4 | 22.26 | 1.35 | 5.48 | 11.47 | 0.42 | 2.77 | 27.59 |
| plantation | 1 | 5 | 1700 | 0.4-0.6 | 21.49 | 1.42 | 5.75 | 5.93 | 0.27 | 2.77 | 22.20 |
| plantation | 1 | 5 | 1700 | 0.6-1 | 21.45 | 1.49 | 5.44 | 4.07 | 0.22 | 3.19 | 18.53 |
| plantation | 2 | 5 | 1680 | 0-0.1 | 21.92 | 1.02 | 5.59 | 23.55 | 0.92 | 2.88 | 25.59 |
| plantation | 2 | 5 | 1680 | 0.1-0.2 | 23.37 | 1.06 | 5.54 | 14.03 | 1.15 | 2.30 | 12.17 |
| plantation | 2 | 5 | 1680 | 0.2-0.4 | 22.53 | 1.17 | 5.53 | 6.28 | 0.40 | 2.38 | 15.86 |
| plantation | 2 | 5 | 1680 | 0.4-0.6 | 23.29 | 1.20 | 6.13 | 3.03 | 0.33 | 1.94 | 9.08 |
| plantation | 2 | 5 | 1680 | 0.6-1 | 24.47 | 1.24 | 5.99 | 2.36 | 0.28 | 1.94 | 8.37 |
| plantation | 3 | 5 | 1720 | 0-0.1 | 35.33 | 0.92 | 5.05 | 49.11 | 2.05 | 7.70 | 23.95 |
| plantation | 3 | 5 | 1720 | 0.1-0.2 | 30.61 | 1.12 | 5.16 | 17.01 | 0.90 | 3.40 | 18.94 |
| plantation | 3 | 5 | 1720 | 0.2-0.4 | 27.55 | 1.17 | 5.23 | 14.55 | 1.00 | 3.45 | 14.58 |
| plantation | 3 | 5 | 1720 | 0.4-0.6 | 27.14 | 1.27 | 5.25 | 8.87 | 0.94 | 2.56 | 9.49 |
| plantation | 3 | 5 | 1720 | 0.6-1 | 27.42 | 1.38 | 5.39 | 5.04 | 0.59 | 3.29 | 8.58 |
| plantation | 16 | 11 | 1600 | 0-0.1 | 26.57 | 0.90 | 4.56 | 41.24 | 1.88 | 1.26 | 21.90 |
| plantation | 16 | 11 | 1600 | 0.1-0.2 | 28.96 | 1.06 | 4.70 | 21.87 | 1.11 | 0.88 | 19.75 |
| plantation | 16 | 11 | 1600 | 0.2-0.4 | 30.67 | 1.13 | 4.99 | 16.37 | 0.95 | 0.67 | 17.23 |
| plantation | 16 | 11 | 1600 | 0.4-0.6 | 27.34 | 1.25 | 5.15 | 11.87 | 0.83 | 0.98 | 14.24 |
| plantation | 16 | 11 | 1600 | 0.6-1 | 26.77 | 1.27 | 4.96 | 7.45 | 0.64 | 0.50 | 11.56 |
| plantation | 17 | 11 | 1650 | 0-0.1 | 30.75 | 0.87 | 5.25 | 40.86 | 1.99 | 1.34 | 20.54 |
| plantation | 17 | 11 | 1650 | 0.1-0.2 | 31.18 | 0.97 | 5.37 | 17.54 | 1.25 | 1.12 | 13.99 |
| plantation | 17 | 11 | 1650 | 0.2-0.4 | 40.87 | 1.00 | 5.49 | 14.09 | 1.02 | 0.71 | 13.84 |
| plantation | 17 | 11 | 1650 | 0.4-0.6 | 25.97 | 1.20 | 5.67 | 13.71 | 0.90 | 0.58 | 15.23 |
| plantation | 17 | 11 | 1650 | 0.6-1 | 25.28 | 1.17 | 5.61 | 5.19 | 0.61 | 0.43 | 8.55 |
| plantation | 18 | 11 | 1630 | 0-0.1 | 31.49 | 0.84 | 5.33 | 34.79 | 1.76 | 1.16 | 19.73 |
| plantation | 18 | 11 | 1630 | 0.1-0.2 | 31.34 | 0.96 | 5.41 | 27.76 | 1.68 | 0.80 | 16.48 |
| plantation | 18 | 11 | 1630 | 0.2-0.4 | 31.38 | 1.05 | 5.44 | 16.45 | 1.14 | 0.66 | 14.41 |
| plantation | 18 | 11 | 1630 | 0.4-0.6 | 36.62 | 1.07 | 5.51 | 8.45 | 0.78 | 0.46 | 10.87 |
| plantation | 18 | 11 | 1630 | 0.6-1 | 31.93 | 1.12 | 5.54 | 6.71 | 0.68 | 0.47 | 9.80 |
| plantation | 11 | 13 | 1700 | 0-0.1 | 34.03 | 0.78 | 5.33 | 58.94 | 3.22 | 2.78 | 18.30 |
| plantation | 11 | 13 | 1700 | 0.1-0.2 | 31.48 | 0.91 | 5.46 | 33.33 | 2.05 | 1.44 | 16.30 |
| plantation | 11 | 13 | 1700 | 0.2-0.4 | 25.55 | 1.01 | 5.79 | 13.57 | 1.07 | 0.67 | 12.73 |
| plantation | 11 | 13 | 1700 | 0.4-0.6 | 25.86 | 1.06 | 5.99 | 9.13 | 0.97 | 0.60 | 9.37 |
| plantation | 11 | 13 | 1700 | 0.6-1 | 26.22 | 1.13 | 5.55 | 5.25 | 0.64 | 0.56 | 8.24 |
| plantation | 12 | 13 | 1710 | 0-0.1 | 26.30 | 0.91 | 5.42 | 43.52 | 2.36 | 1.18 | 18.43 |
| plantation | 12 | 13 | 1710 | 0.1-0.2 | 27.43 | 0.98 | 5.48 | 27.36 | 1.65 | 1.53 | 16.59 |
| plantation | 12 | 13 | 1710 | 0.2-0.4 | 24.56 | 1.08 | 5.53 | 20.46 | 1.25 | 0.79 | 16.33 |
| plantation | 12 | 13 | 1710 | 0.4-0.6 | 21.99 | 1.36 | 5.65 | 7.35 | 0.75 | 0.71 | 9.83 |
| plantation | 12 | 13 | 1710 | 0.6-1 | 19.90 | 1.29 | 5.76 | 4.78 | 0.46 | 1.34 | 10.34 |
| plantation | 13 | 13 | 1725 | 0-0.1 | 26.76 | 1.09 | 5.39 | 23.83 | 1.54 | 2.68 | 15.48 |
| plantation | 13 | 13 | 1725 | 0.1-0.2 | 23.51 | 1.22 | 5.41 | 13.05 | 0.93 | 1.94 | 14.08 |
| plantation | 13 | 13 | 1725 | 0.2-0.4 | 19.82 | 1.41 | 5.47 | 10.92 | 0.83 | 1.44 | 13.21 |
| plantation | 13 | 13 | 1725 | 0.4-0.6 | 22.96 | 1.34 | 5.47 | 10.91 | 0.76 | 1.04 | 14.27 |
| plantation | 13 | 13 | 1725 | 0.6-1 | 23.41 | 1.35 | 5.34 | 9.01 | 0.74 | 1.59 | 12.11 |
| plantation | 22 | 20 | 1530 | 0-0.1 | 20.51 | 1.14 | 4.85 | 21.62 | 1.20 | 3.91 | 17.94 |
| plantation | 22 | 20 | 1530 | 0.1-0.2 | 21.75 | 1.28 | 4.84 | 13.47 | 0.91 | 2.89 | 14.87 |
| plantation | 22 | 20 | 1530 | 0.2-0.4 | 19.81 | 1.22 | 4.47 | 9.70 | 0.78 | 4.19 | 12.47 |
| plantation | 22 | 20 | 1530 | 0.4-0.6 | 22.03 | 1.25 | 4.66 | 6.46 | 0.52 | 3.45 | 12.46 |
| plantation | 22 | 20 | 1530 | 0.6-1 | 22.13 | 1.26 | 5.10 | 4.38 | 0.46 | 2.89 | 9.56 |
| plantation | 23 | 20 | 1518 | 0-0.1 | 20.72 | 1.19 | 4.86 | 16.50 | 0.95 | 2.70 | 17.37 |
| plantation | 23 | 20 | 1518 | 0.1-0.2 | 19.21 | 1.31 | 4.83 | 13.36 | 0.90 | 3.45 | 14.78 |
| plantation | 23 | 20 | 1518 | 0.2-0.4 | 17.70 | 1.40 | 4.71 | 10.08 | 0.64 | 4.37 | 15.85 |
| plantation | 23 | 20 | 1518 | 0.4-0.6 | 23.36 | 1.41 | 4.64 | 5.10 | 0.42 | 4.56 | 12.05 |
| plantation | 23 | 20 | 1518 | 0.6-1 | 17.90 | 1.51 | 4.88 | 3.74 | 0.34 | 3.63 | 10.93 |
| plantation | 24 | 20 | 1510 | 0-0.1 | 19.84 | 1.11 | 4.61 | 20.92 | 1.15 | 2.70 | 18.22 |
| plantation | 24 | 20 | 1510 | 0.1-0.2 | 16.05 | 1.44 | 4.47 | 14.36 | 0.92 | 2.89 | 15.56 |
| plantation | 24 | 20 | 1510 | 0.2-0.4 | 13.91 | 1.42 | 4.40 | 9.91 | 0.64 | 3.63 | 15.37 |
| plantation | 24 | 20 | 1510 | 0.4-0.6 | 19.28 | 1.45 | 4.81 | 5.36 | 0.45 | 3.26 | 11.96 |
| plantation | 24 | 20 | 1510 | 0.6-1 | 20.23 | 1.40 | 4.86 | 3.75 | 0.39 | 3.45 | 9.61 |
| plantation | 25 | 26 | 1600 | 0-0.1 | 18.71 | 1.35 | 5.93 | 21.22 | 1.22 | 4.56 | 17.38 |
| plantation | 25 | 26 | 1600 | 0.1-0.2 | 19.49 | 1.36 | 6.12 | 17.79 | 1.05 | 4.37 | 16.90 |
| plantation | 25 | 26 | 1600 | 0.2-0.4 | 20.60 | 1.29 | 6.16 | 14.89 | 0.96 | 4.93 | 15.58 |
| plantation | 25 | 26 | 1600 | 0.4-0.6 | 22.75 | 1.09 | 6.15 | 9.26 | 0.85 | 3.82 | 10.84 |
| plantation | 25 | 26 | 1600 | 0.6-1 | 23.42 | 1.11 | 5.52 | 7.52 | 0.77 | 5.11 | 9.72 |
| plantation | 26 | 26 | 1640 | 0-0.1 | 21.21 | 1.09 | 5.68 | 25.17 | 1.52 | 5.67 | 16.52 |
| plantation | 26 | 26 | 1640 | 0.1-0.2 | 22.33 | 1.11 | 6.07 | 18.42 | 1.20 | 5.85 | 15.38 |
| plantation | 26 | 26 | 1640 | 0.2-0.4 | 23.18 | 1.13 | 6.06 | 14.08 | 1.03 | 4.93 | 13.65 |
| plantation | 26 | 26 | 1640 | 0.4-0.6 | 22.44 | 1.15 | 6.00 | 9.54 | 0.93 | 4.56 | 10.27 |
| plantation | 26 | 26 | 1640 | 0.6-1 | 22.27 | 1.23 | 5.95 | 4.93 | 0.58 | 3.45 | 8.46 |
| plantation | 27 | 26 | 1550 | 0-0.1 | 19.25 | 1.19 | 5.52 | 21.11 | 1.26 | 3.63 | 16.82 |
| plantation | 27 | 26 | 1550 | 0.1-0.2 | 20.31 | 1.21 | 5.65 | 11.92 | 0.89 | 3.82 | 13.34 |
| plantation | 27 | 26 | 1550 | 0.2-0.4 | 21.31 | 1.17 | 5.64 | 8.69 | 0.80 | 4.46 | 10.91 |
| plantation | 27 | 26 | 1550 | 0.4-0.6 | 21.55 | 1.18 | 5.74 | 5.46 | 0.65 | 4.56 | 8.45 |
| plantation | 27 | 26 | 1550 | 0.6-1 | 21.66 | 1.20 | 5.69 | 3.94 | 0.57 | 3.08 | 6.89 |
| plantation | 19 | 54 | 1500 | 0-0.1 | 30.58 | 0.82 | 4.77 | 40.78 | 2.50 | 5.11 | 16.29 |
| plantation | 19 | 54 | 1500 | 0.1-0.2 | 30.52 | 0.97 | 4.84 | 33.37 | 1.70 | 7.15 | 19.61 |
| plantation | 19 | 54 | 1500 | 0.2-0.4 | 30.26 | 0.90 | 5.02 | 23.83 | 1.37 | 6.04 | 17.39 |
| plantation | 19 | 54 | 1500 | 0.4-0.6 | 30.60 | 0.91 | 4.95 | 15.88 | 0.94 | 4.93 | 16.86 |
| plantation | 19 | 54 | 1500 | 0.6-1 | 30.66 | 0.88 | 5.02 | 16.16 | 1.02 | 7.71 | 15.91 |
| plantation | 20 | 54 | 1518 | 0-0.1 | 26.52 | 0.85 | 4.64 | 33.74 | 2.27 | 9.56 | 14.87 |
| plantation | 20 | 54 | 1518 | 0.1-0.2 | 27.23 | 0.91 | 4.68 | 23.82 | 1.67 | 10.95 | 14.30 |
| plantation | 20 | 54 | 1518 | 0.2-0.4 | 27.48 | 0.93 | 4.83 | 16.80 | 1.06 | 12.52 | 15.81 |
| plantation | 20 | 54 | 1518 | 0.4-0.6 | 27.81 | 0.98 | 5.00 | 8.74 | 0.72 | 11.23 | 12.13 |
| plantation | 20 | 54 | 1518 | 0.6-1 | 27.90 | 1.00 | 5.07 | 8.57 | 0.64 | 11.23 | 13.45 |
| plantation | 21 | 54 | 1480 | 0-0.1 | 26.21 | 0.87 | 4.83 | 35.94 | 2.20 | 14.93 | 16.34 |
| plantation | 21 | 54 | 1480 | 0.1-0.2 | 28.91 | 0.91 | 4.66 | 26.40 | 1.62 | 10.11 | 16.35 |
| plantation | 21 | 54 | 1480 | 0.2-0.4 | 27.44 | 0.97 | 4.84 | 19.75 | 1.23 | 9.56 | 16.07 |
| plantation | 21 | 54 | 1480 | 0.4-0.6 | 28.40 | 0.94 | 4.95 | 11.24 | 0.79 | 7.89 | 14.16 |
| plantation | 21 | 54 | 1480 | 0.6-1 | 28.33 | 0.98 | 5.13 | 9.85 | 0.73 | 9.56 | 13.53 |
| SCF | 31 | 60 | 1720 | 0-0.1 | 23.94 | 1.05 | 4.73 | 34.62 | 1.83 | 8.91 | 18.93 |
| SCF | 31 | 60 | 1720 | 0.1-0.2 | 27.81 | 1.04 | 4.78 | 23.60 | 1.29 | 7.98 | 18.33 |
| SCF | 31 | 60 | 1720 | 0.2-0.4 | 21.97 | 1.17 | 4.85 | 15.16 | 0.89 | 6.68 | 16.98 |
| SCF | 31 | 60 | 1720 | 0.4-0.6 | 20.68 | 1.36 | 4.99 | 6.09 | 0.63 | 5.93 | 9.68 |
| SCF | 31 | 60 | 1720 | 0.6-1 | 22.13 | 1.44 | 5.13 | 5.06 | 0.60 | 5.19 | 8.46 |
| SCF | 32 | 60 | 1600 | 0-0.1 | 15.81 | 1.25 | 4.58 | 20.43 | 0.98 | 4.44 | 20.90 |
| SCF | 32 | 60 | 1600 | 0.1-0.2 | 19.21 | 1.22 | 4.66 | 11.57 | 0.71 | 3.51 | 16.39 |
| SCF | 32 | 60 | 1600 | 0.2-0.4 | 19.89 | 1.40 | 4.68 | 7.20 | 0.64 | 3.70 | 11.25 |
| SCF | 32 | 60 | 1600 | 0.4-0.6 | 18.02 | 1.56 | 4.94 | 5.33 | 0.43 | 3.89 | 12.28 |
| SCF | 32 | 60 | 1600 | 0.6-1 | 22.07 | 1.51 | 4.95 | 3.00 | 0.43 | 3.14 | 7.03 |
| SCF | 33 | 60 | 1690 | 0-0.1 | 23.87 | 1.08 | 4.54 | 27.58 | 1.37 | 8.54 | 20.19 |
| SCF | 33 | 60 | 1690 | 0.1-0.2 | 26.02 | 1.21 | 4.77 | 17.61 | 1.10 | 4.63 | 16.01 |
| SCF | 33 | 60 | 1690 | 0.2-0.4 | 25.43 | 1.29 | 4.91 | 11.38 | 0.86 | 5.93 | 8.46 |
| SCF | 33 | 60 | 1690 | 0.4-0.6 | 23.88 | 1.46 | 5.01 | 6.40 | 0.69 | 3.33 | 7.03 |
| SCF | 33 | 60 | 1690 | 0.6-1 | 24.78 | 1.51 | 4.98 | 5.69 | 0.59 | 5.37 | 9.70 |
